# Hyperspectral Imaging for Determining Pigment Contents in Cucumber Leaves in Response to Angular Leaf Spot Disease

**DOI:** 10.1038/srep27790

**Published:** 2016-06-10

**Authors:** Yan-Ru Zhao, Xiaoli Li, Ke-Qiang Yu, Fan Cheng, Yong He

**Affiliations:** 1 College of Biosystems Engineering and Food Science, Zhejiang University, 866 Yuhangtang Road, Hangzhou 310058, China; 2 College of Mechanical and Electronic Engineering, Northwest A&F University, Yangling 712100, China; 3 Institute of Biotechnology, Zhejiang University, 866 Yuhangtang Road, Hangzhou 310058, China

## Abstract

Hyperspectral imaging technique was employed to determine spatial distributions of chlorophyll (*Chl*), and carotenoid (*Car*) contents in cucumber leaves in response to angular leaf spot (ALS). Altogether, 196 hyperspectral images of cucumber leaves with five infection severities of ALS were captured by a hyperspectral imaging system in the range of 380–1,030 nm covering 512 wavebands. Mean spectrum were extracted from regions of interest (ROIs) in the hyperspectral images. Partial least square regression (PLSR) models were used to develop quantitative analysis between the spectra and the pigment contents measured by biochemical analyses. In addition, regression coefficients (RCs) in PLSR models were employed to select important wavelengths (IWs) for modelling. It was found that the PLSR models developed by the IWs provided the optimal measurement results with correlation coefficient (*R*) of prediction of 0.871 and 0.876 for *Chl* and *Car* contents, respectively. Finally, *Chl* and *Car* distributions in cucumber leaves with the ALS infection were mapped by applying the optimal models pixel-wise to the hyperspectral images. The results proved the feasibility of hyperspectral imaging for visualizing the pigment distributions in cucumber leaves in response to ALS.

Cucumber (*Cucumis sativus*) has always been regarded as a widely cultivated plant in the world. FAO reported that the cucumber fruits production of the world reached to 60,895,225 tons in 2012^1^. However, cucumbers often suffer from the invasion of various diseases, which affect the yield and quality of the cucumber fruits. Angular leaf spot (ALS) caused by the bacterium *Pseudomonas syringae* pv. *lachrymans* may severely affect cucumber growth by foliar infections. The bacterium firstly causes small, water-soaked lesions on the abaxial leaf surface; then, these infected areas turn brown or straw-colored^2^. Mesophyll tissue affected by bacterial infection may show increased respiration, disintegration or collapse of cells, and degradation of pigments such as chlorophyll (*Chl*) and carotenoid (*Car*)^3^. The bacterial infection not only causes the reduction of pigments, but also results in spatial heterogeneity of leaf pigments. *Chl* is the most important pigment in plants and its concentration controls photosynthetic potential and primary production. *Car* is the second major group of plant pigments, and its concentration provides much complementary information on vegetation physiological status^4^. *Chl* and *Car* contents have been successfully used for diagnosing physiological state of plants under disease stresses^5^,^6^,^7^. However, no research endeavors have been reported yet for characterizing the spatial distribution of pigments within the leaves infected by bacterium. Therefore, developing efficient methods for quantitative analysis of pigment contents and exploring pigment distributions in space are significant for the diagnosis of mild diseases. Detection of abnormal spots with lower *Chl* and *Car* values in leaves will be especially meaningful for detecting mild ALS diseases.

Ultraviolet spectrophotometry and high performance liquid chromatography have been used to determine *Chl* and *Car* contents through a series of operations including weighting, grinding, extracting supernatant, and measuring^8^,^9^. However, these methods involve labor-intensive, time-consuming and tedious extraction procedures. The chlorophyll meter (SPAD-502), a simple and portable diagnostic tool, can measure the greenness or relative *Chl* content by acquiring the absorbance of leaves in the red and near infrared (NIR) regions at individual points in a non-destructive way^10^. However, the chlorophyll meter method is an impractical tool for a large-scale real-time plants monitoring under filed conditions. During the past few decades, spectroscopic techniques have been used extensively as powerful and effective analytical tools for estimating *Chl* and *Car* content in leaves^11^,^12^,^13^. Previous literatures confirmed that spectral reflectance of vegetation in the visible region was primarily governed by pigments^14^,^15^,^16^. Unfortunately, spectroscopic technique is often used to measure the averaged spectrum of the sample which fails to provide the spectral details at each pixel on gray-scale images of the targets. However, spectrum information with high spatial resolution is vital to detect the disease infection at early stage.

Hyperspectral imaging technique combines reflectance spectra with image processing. It has promoted recent progress in remote sensing and has been widely used for mapping the distribution of physiological characteristics in plants^17^. Yu *et al*.^18^ mapped spatial distribution of nitrogen in whole pepper plant with characteristic hyperspectral images. Shi *et al*.^19^ investigated chlorophyll concentration distribution to determine nitrogen deficiency in cucumber plants by using hyperspectral imaging combined with chemometrics. Zhang *et al*.^20^ estimated nitrogen (N), phosphorus (P), and potassium (K) distributions in oilseed rape leaves by applying the optimal calibration models in each pixel of the reduced hyperspectral images. A majority of studies focusing on the disease detection rely on the spectral characteristics of the infected plants based on hyperspectral imaging^21^,^22^,^23^. Hyperspectral imaging is capable of providing spectral details at each pixel in space, which makes it possible to map the pigment distributions in the samples. However, there is little research on mapping *Chl* and *Car* distributions of the plants in response to pathogen infection using hyperspectral imaging.

The aim of this work was to determine the *Chl* and *Car* distributions in the cucumber leaves with bacterial infection at five severities by using the hyperspectral imaging technique. The specific objectives were to: (1) extract important wavelengths (IWs) from hyperspectral data for establishing the optimal quantitative relationships between spectra and pigment contents; (2) establish regression models for predicting *Chl* and *Car* contents and evaluate the performance of these quantitative analyses; (3) visualize *Chl* and *Car* concentration distributions in cucumber leaves in response to ALS based on the selected hyperspectral images.

## Results and Discussion

### Statistics of the pigments measured by biochemical analyses

**Figure 1:**
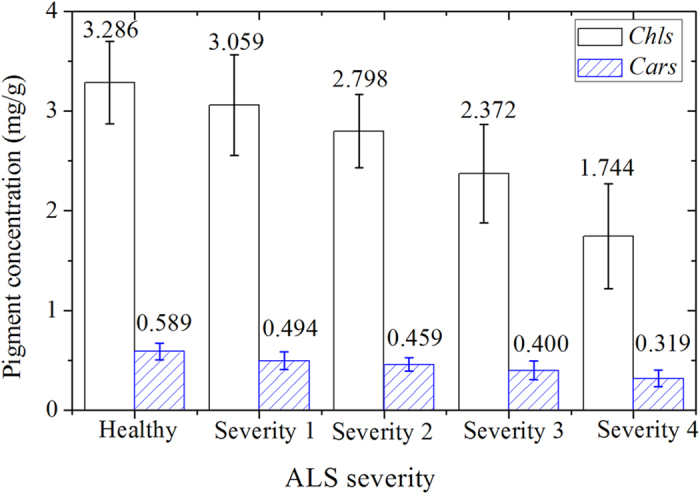
Mean measured *Chl*-BA and *Car*-BA contents in healthy leaves and ALS infected leaves. *Chl* and *Car* contents were determined by biochemical analysis (*Chl*-BA, *Car*-BA). Pigment “contents” values of the healthy and infected leaves were calculated and shown as 10 numbers to represent the variation trend of the pigments with the severity of the ALS disease.

A total of 194 cucumber leaves from healthy and infected plants with four severities of bacterial infection were collected for *Chl* and *Car* content biochemical analyses (*Chl*-BA, *Car*-BA). The statistical analysis results of the measured *Chl*-BA and *Car*-BA contents are shown in Fig. 1. The mean *Chl*-BA and *Car*-BA content were 3.286 mg/g and 0.589 mg/g for healthy plants, respectively. There was an obvious reduction trend of the averaged pigment contents along with the severity of disease, ranging from 3.059 mg/g to 1.744 mg/g for *Chl*-BA, and 0.494 mg/g to 0.319 mg/g for *Car*-BA. As expected, the mean values of *Chl*-BA and *Car*-BA contents displayed a descending tendency with the severities of the bacterial infection in leaves. The descending trends of *Chl*-BA and *Car*-BA content with disease stress in plants also confirmed previous results from the literature^24^.

**Figure 2:**
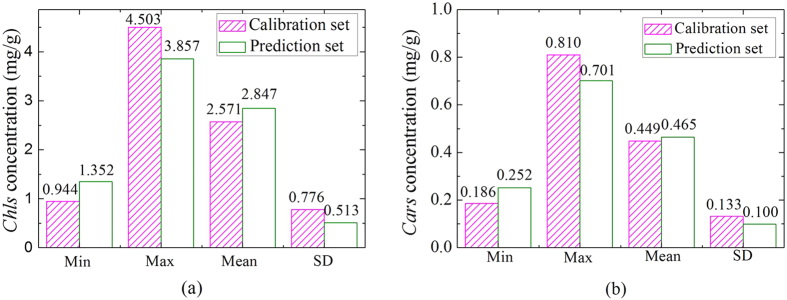
The measured *Chl*-BA (**a**) and *Car*-BA (**b**) content distributions in the calibration set and prediction set. Note: Min: Minimum; Max: Maximum; SD: Standard deviation. SPXY method was used to divide all samples into calibration set with 130 samples and prediction sets with 64 samples for modelling.

The SPXY algorithm^25^ was implemented to separate all samples into a calibration set (130 samples) and a prediction set (64 samples) according to their differences in both *x* (spectra) and *y* (reference value) spaces. Figure 2 shows the statistical results of *Chl*-BA and *Car*-BA contents in calibration set and prediction set. There were evident variations of pigment contents in the calibration set, ranging from 0.944 mg/g to 4.503 mg/g for *Chl*-BA, and from 0.186 mg/g to 0.810 mg/g for *Car*-BA. The ranges of *Chl*-BA and *Car*-BA contents in the prediction set were from 1.352 mg/g to 3.857 mg/g and 0.252 mg/g to 0.701 mg/g, respectively. The calibration set has a lower mean value and a higher standard deviation (SD) than the prediction set. Generally, the calibration set with a wide concentration distribution of the chemical values is conducive to build a stable calibration model^18^.

### Spectral features of leaves

The spectra of whole leaf were averaged as the representative spectrum of a sample, and 194 averaged spectra in the region of 380–1,030 nm were obtained altogether. The starting 18 bands and ending 18 bands were removed to improve signal-noise-ratio (SNR), and wavebands in the range of 400–1,000 nm (476 wavelengths) were used for further processing.

**Figure 3:**
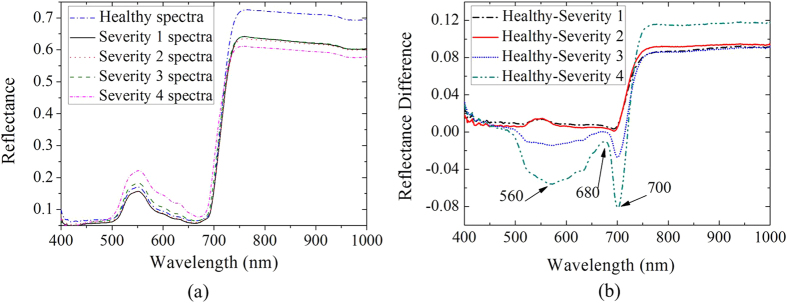
The average spectral reflectance curves of cucumber leaves. (**a**) Average spectral curves of cucumber leaves with five ALS disease severities; (**b**) Subtracted reflectance-differences between these average spectra. Reflectance of healthy samples and samples of four ALS disease severities (n = 40, 39, 39, 37, 39) were averaged respectively, resulting in five curves.

The spectra of the samples at each bacterial infection severity were averaged to analyze spectra variations of all infected leaves and five spectra are shown in Fig. 3(a). All spectra had similar characteristics of the “green plants”^26^,^27^,^28^,^29^,^30^. However, there were obvious reflectance value differences between the spectra of healthy and infected leaves in Fig. 3(a). In visible region (400–780 nm), infected leaves had higher reflectance values than healthy leaves, which was caused by the reduction of *Chl* content in the bacterial infected cucumber leaves^27^. However, in NIR region (780–1,000 nm), reflectance values of infected leaves were lower than those of healthy leaves, which was mainly due to the internal structure damaged caused by the bacteria. Besides, red-edge swing descended and red edge position showed “blue movement” when the green plants suffered diseases stress^31^. Spectra showed an increasing trend in the visible region from severity 1 to severity 4 in infected leaves, which was consistent with the measured *Chl*-BA and *Car*-BA content variations shown in Fig. 1.

However, there were no evident difference in spectra among samples with severity 1, severity 2 and severity 3 ALS infections in Fig. 3(a), which also means that average spectrum of the whole leaf fails to provide spectral details of mild diseases. The obvious differences of these spectral curves were expressed through subtracting spectra of infected samples from healthy leaves spectra. The reflectance-differences (RDs) of the averaged spectra are displayed in Fig. 3(b). The spectra of the infected cucumber leaves were clearly distinguished by the RDs, especially around 560 nm, 680 nm, and 700 nm, which were closely related to the pigments of leaves^32^. The reflectance of pigments in visible region are the theoretical basis for establishing the mathematical correlation between spectral data and compositional contents.

### Partial least squares regression models

**Figure 4:**
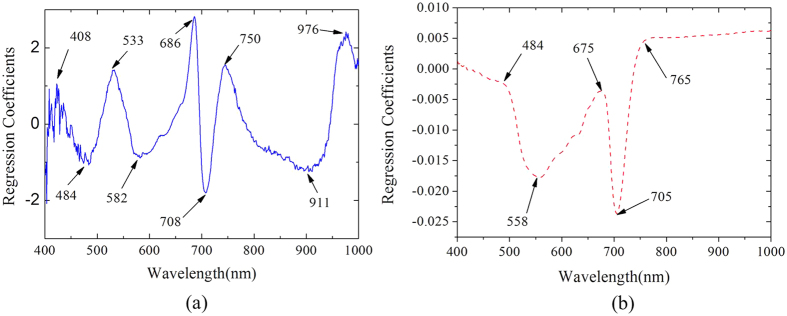
Regression coefficients (RCs) of each wavelength in PLSR based on the whole spectra. (**a**) RCs of each wavelength for *Chl*-HSI; (**b**) RCs of each wavelength for *Car*-HSI. The local extremum values of regression coefficient represent the importance of each wavelength. The selected important wavelengths which carried effective information were employed to establish the regression models to improve the speed of the modelling.

As shown in Fig. 4(a), nine wavelengths are selected as the important wavelengths (IWs) to predict the *Chl* using hyperspectral imaging (*Chl*-HSI) model. Among the selected wavelengths, seven wavelengths at 408, 484, 533, 582, 686, 708, and 750 nm were located in the visible region, which confirmed that pigments primarily governed the spectral reflectance of vegetation in the visible region^16^. In addition, 911 and 976 nm, which were close to the second overtones O-H stretching, were sensitive to water status of leaves. ALS destroys the cellular integrity of the cucumber tissue and may result in changes of the relative water content^33^. Figure 4(b) shows the RCs of each wavelength and five wavelengths at 484, 558, 675, 705, and 765 nm were selected as the IWs for predicting *Car* by hyperspectral imaging (*Car*-HSI). There are some similar IWs for both *Chl* and *Car*, which explained by the similar optical properties of *Chl* and *Car*.

The PLSR algorithm was employed to establish regression models for evaluating *Chl*-HSI and *Car*-HSI contents in the cucumber leaves based on the selected IWs and whole spectra, respectively. The results of the PLSR models are enumerated in Table 1. The best performance of Model 1 and Model 3 were achieved with *Rc* of 0.913, *Rp* of 0.870, RMSEC of 0.315, and RMSEP of 0.255 for *Chl*-HSI content estimation, *Rc* of 0.796, *Rp* of 0.886, RMSEC of 0.080, and RMSEP of 0.049 for *Car*-HSI contents prediction. However, Model 1 and Model 3 with 476 variables involved in the modeling were time-consuming and failed to offer the simplified regression functions for predicting *Chl*-HSI and *Car*-HSI contents. Model 2 and Model 4 also provided similar results with *Rc* of 0.901, *Rp* of 0.871, RMSEC of 0.355, and RMSEP of 0.250 for *Chl*-HSI content, *Rc* of 0.803, *Rp* of 0.876, RMSEC of 0.079, and RMSEP of 0.050 for *Car*-HSI content. Although 98.32% (476 vs. 9) of the variables in Model 2 were eliminated, *Rc* of Model 2 only showed a slight reduction from 0.913 to 0.901, and *Rp* had a similar performance with an increase from 0.870 in Model 1 to 0.871. Similar results obtained in Model 4. Moreover, Model 4 with only five wavelengths improved the modeling speed. Therefore, Model 2 and Model 4 were promising to predict *Chl*-HSI and *Car*-HSI contents of the cucumber leaves.

From Models 2 and 4, the quantitative relationships between the spectral reflectance and pigments contents are shown in function (1) and (2):


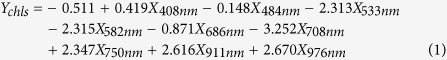



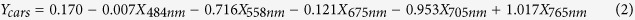


where, *Y* is the predicted pigment content, *X*_*i* nm_ is the reflectance spectra at the selected IWs.

As shown in Table 1, Model 2 and Model 4 were the optimal models to estimate *Chl*-HSI and *Car*-HSI contents. Therefore, the multi-linear functions (1) and (2) were capable to calculate the *Chl*-HSI and *Car*-HSI contents at each pixel point of the hyperspectral images of the samples.

### Distribution maps of pigments in cucumber leaves

**Figure 5:**
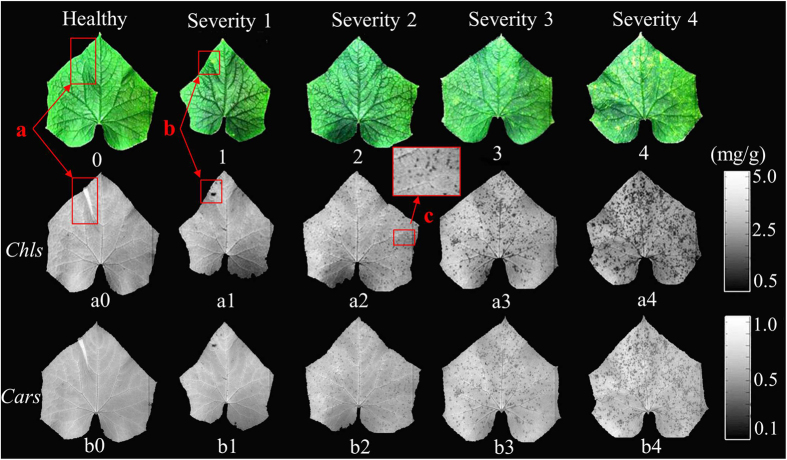
*Chl*-HSI and *Car*-HSI distributions in cucumber leaves with the ALS infection. *Chl*-HSI and *Car*-HSI of leaves in hyperspectral images were calculated based on the function (2) and (3), and the spatial distribution was executed by an image processing program. 0–4: RGB (R: 662 nm; G: 554 nm; B: 450 mn) images of healthy leaves and samples with disease severity 1, 2, 3, and 4, respectively; a0–a4: *Chl*-HSI content distributions of the samples; b0–b4: *Car*-HSI content visualization maps of the cucumber leaves; a: wrinkle part; b: disease spot; c: partially enlarged details of the disease spots.

The optimal simplified models (functions (1) and (2)) were employed to estimate *Chl*-HSI and *Car*-HSI values at each pixel of the hyperspectral images. Then, *Chl*-HSI and *Car*-HSI distribution maps in the cucumber leaves were generated by a developed image processing program. Figure 5 shows the *Chl*-HSI and *Car*-HSI content distributions in the cucumber leaves in response to ALS. Two gray-scale color bars (0–255 levels) were generated with different *Chl*-HSI and *Car*-HSI values from small to large shown in different color from black (0 level) to white (255 level).

RGB images of the cucumber leaves with five severity stages are shown in Fig. 5(0–4). There were no obvious symptoms appeared on the upper side of the leaves in Fig. 5(0–2), therefore it was difficult to diagnose the bacterial early infection according to the RGB images. However, several yellow points appeared on the upper side of the cucumber leaves with the bacterial infection at severity 3 and severity 4, such as Fig. 5(3,4). The color difference between the infected area and the non-infected region was not evident. Overall, it was not easy to estimate the epidemic situation according to the RGB images of the samples. Figure 5(a0–a4,b0–b4) show the *Chl*-HSI and *Car*-HSI distribution maps in the cucumber leaves. More and more disease spots with low *Chl*-HSI and *Car*-HSI contents appeared with the increasing severity of the bacterial infection. *Chl*-HSI values of the whole healthy leaf were homogeneous, and there was no abnormal point in Fig. 5(a0) except for the (a) region, which was caused by the wrinkle part. The points with lower *Chl*-HSI content than other parts were clearly displayed at (b) point in Fig. 5(a1). However, this point is hard to be detected at (b) region in Fig. 5(1). Furthermore, several small disease spots are displayed in Fig. 5(a2), as illustrated by the partially enlarged details of the disease spots in (c) region. However, there was no obvious disease spots appeared in Fig. 5(2). It could be concluded that the disease spot with lower *Chl*-HSI content was easily detected in the *Chl*-HSI distribution maps at early stage. More disease spots with low *Chl*-HSI content are shown in Fig. 5(a3,a4). Meanwhile, Fig. 5(b0–b5) show the *Car*-HSI content distributions in the cucumber leaves. It could be found that *Car*-HSI content had similar variations with the *Chl*-HSI content in the cucumber leaves in response to the ALS infection. *Chl* and *Cars* are both light-harvesting pigments, but *Chl* is the most abundant and critical pigment for photosynthesis. Therefore, *Chl*-HSI distributions had better performance than *Car*-HSI distributions for detecting ALS severities.

Disease symptoms commonly reflect the interaction between plant and pathogen. Once bacterial pathogen successfully infects the leaves, a series of physiological changes would turn up during the process. Infected points firstly would appear water soaked symptom, pigments break would be then broken down and the infected area would become straw colored. Finally, the pathogen would cause wilting, browning, and death of leaves^3^. When the cucumber leaves were attacked by the *Pseudomonas syringae* pv. *lachrymans*, water soaked small disease spots firstly appeared on the underside of leaves. However, these small disease spots can’t be easily detected from the upper surface by naked eyes. More disease spots appeared on the upper side of the leaves with the increasing of disease severity. The variation of pigments in infected cells is one of the most important characteristics associated with the diseases severity of plants. As shown in Fig. 5, the visualization of *Chl* and *Car* spatial distribution are helpful and meaningful to observe the ALS disease severities.

Besides the bacterial infection, pigment disorder of leaves also could be caused by fungal, viral infections, insect, and nutrient deficiencies. For example, nitrogen deficiency could cause a low chlorophyll density distribution on leaves. In this case, however, the symptom is usually stabilized, and would never spread over infected time. Also, some insects could cause significant damage by eating mesophyll^34^. Nevertheless, the destroyed area always express as a worm eaten path or wormholes which contain no pigment. These characteristics of both stresses were mostly different from that of diseases. Leaf spot is regarded as one of the most common fungal and bacterial plant symptoms. Generally, shapes, sizes, and colors of leaf spots caused by various pathogens are different^35^, and these physical features would change with the severity degree or incubation stage of infection. In addition, many microbial diseases would spread over a large area in groves and plantations through accidental introduction of vectors or through infected plant materials^36^. The specifics of various symptoms change the spectral and spatial information of plants. Thereby, hyperspectral imaging has the advantage for real-time detection of plant disease at early stage by mapping the pigment spatial distribution or revealing the physical specificities of diseases. In future work, more kinds of diseases with different severities or infection incubation should be carried out to develop more adequate models on plants’ pigment images production. More attention should be paid on the statistical analysis of physical specifics (shape, infection size and others) of various diseases in future investigation.

## Conclusions

In summary, this study shows that the hyperspectral imaging coupled with chemometrics is an effective method to measure *Chl*-HSI and *Car*-HSI contents and to generate distribution maps of pigments in cucumber leaves with the ALS infection. The PLSR models with nine wavelengths at 408, 484, 533, 582, 686, 708, 750, 911, 976 nm and five wavelengths at 484, 558, 675, 705, and 765 nm could provide better accuracy than the whole-PLSR models for detecting *Chl*-HSI and *Car*-HSI contents of cucumber leaves. In addition, ALS disease spots were shown clearly in the *Chl*-HSI and *Car*-HSI distribution maps. Applying important wavelengths to detect mild disease from mapping pigment spatial distribution is critical for sensor’s real time assessment.

## Materials and Methods

### Samples

A total of 100 cucumber plants (*Cucumis sativus* L, Jingyu, Tianjin, China) were grown in a greenhouse at Zhejiang University, Hangzhou, China. Twenty uninoculated plants kept as a control group with non-inoculated. *Pseudomonas syringae* pv. *lachrymans*, obtained from Institute of Biotechnology, was inoculated onto cucumber plants at 6-leaf stage as described in the literature^37^. The samples were picked two weeks later after inoculation. The disease severity was determined by the severity and number of disease spots on a cucumber leaf by agriculture experts, and the following five ALS severity groups were defined:

1. non-diseased plants in which no symptoms were observed on the leaves (40 samples);
2. infected plants at severity 1 in which initial symptoms were seen with less than 2 disease spots on one leaf (39 samples);
3. infected plants at severity 2 in which symptoms were a little more severe than stage 1 with 2~4 disease spots on the leaf (39 samples);
4. infected plants at severity 3 with 5~9 disease spots on one leaf (37 samples);
5. infected plants at severity 4 with more than 10 disease spots on one leaf (39 samples).

In this study, a total of 194 samples with five different disease severities were picked in an hour. The tested samples were immediately enclosed into individual plastic bags to keep fresh in incubator with crushed ice around to maintain the constant temperature of 2–4 °C and transported to the laboratory within 2 hours for capturing hyperspectral images.

### Equipment

The hyperspectral images of cucumber leaves were captured by a line-scanning hyperspectral imaging system (380–1030 nm) in reflectance mode. The system is composed of a spectrograph (ImSpectorV10, Spectral Imaging Ltd., Finland), a CCD camera (C8484-05G, Hamamatsu, Hamamatsu) coupled with a lens (OLES23; Specim, Spectral Imaging Ltd., Oulu, Finland), an illumination system with two 150W quartz tungsten halogen lamps (Fiber-Lite DC950 Illuminator, Dolan Jenner Industries Inc., USA) adjusted at an angle of 45° to illuminate the camera’s field of view (FOV), a conveyer operated by a stepper motor (IRCP0076, Isuzu Optics Crop, Taiwan), and a computer with data acquisition and preprocessing software (Spectral-Cube data acquisition V10 software). The spectral resolution is 2.8 nm in 380–1030 nm, and CCD camera has a resolution of 672 × 512 pixels. The distance between samples and the lens was 485 mm, the speed of conveyor was set as 3.8 mm/s, and the exposure time was 0.09s during the image acquisition. To acquire a three-dimension hypercube, each sample was placed on a black background which had a very low reference. The upper side of the cucumber leaf was faced to the camera.

### Image processing

Raw hyperspectral image (*I*_*0*_) was calibrated by white (*W*) and dark (*B*) reference images. The white image was acquired from a standard Teflon tile (~99.9% reflectance); the dark image (~0% reflectance) was obtained by turning off the light source, completely covering the camera leans with its opaque cap and recording the completely response^38^. The calibrated image (*I*) was calculated by the following equation:


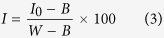


A whole cucumber leaf was selected as a region of interest (ROI) of a sample. A spectral matrix of 196 samples ×512 bands was generated. There are many approaches to execute the target image segmentation from hyperspectral images, such as “Manual Mask” and “Automatic Mask”^39^,^40^. A simple threshold segmentation based on a single band grayscale image was finished with the aid of Environment Visualizing Images (ENVI) softwares (ITT Visual Information, Solutins, USA). In addition, this method is also effective in MATLAB R2009a (The MathWorks, Inc., Natick, MA, USA).

### Biochemical analyses

After the collection of hyperspectral images, samples were immediately cut into pieces and weighted to execute chemical analysis. Each measurement was performed in three replications. Each sample with 0.1 g fresh weight leaf tissue without leaf vein was immersed using a mixture (20 ml) of 80% acetone and 100% ethanol (1:1) for 24 hours in the dark to extract the pigments. *Chl*-BA and *Car*-BA contents were measured by 752UV/Vis spectrophotometry (Inesa Instrument, Shanghai, China) and calculated per unit fresh mass basis with the published methods^41^,^42^:


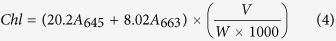



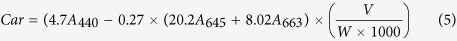


where, *Chl* content was the sum of contents of *Chl* a and *Chl* b; *V* was the volume of the mixed solvent (ml); *W* was the fresh weight of the leaf that was measured (g); *A*_440_ was the absorbance value of the extract solution at 440 nm, *A*_645_ was the absorbance value of the extract solution at 645 nm, *A*_663_ was the absorbance value of the extract solution at 663 nm.

### Data Analysis

Regression coefficients (RCs) of partial least squares regression (PLSR) can be used to measure the association between each variable (*X*) and the response (*Y*)^43^. Generally, variables with small absolute value of RCs can be eliminated and RCs is an effective method to select important wavelengths. PLSR is a statistic method based on multiple linear and stepwise regressions and especially suitable for modeling when the number of variables is greater than the number of samples, and when there is colinearity between variables^44^. In addition, the number of latent variables (LVs) of the PLSR model was based on the lowest value of predicted residual errors sum of squares to avoid over-fitting or under-fitting problems. PLSR was applied to construct the calibration models with the aid of Unscrambler X10.1 (CAMO Process As, Oslo, Norway). It combines the most useful information from hundreds of bands into the first several factors to develop the calibration model. Leave-one-out cross-validation was applied to validate the performance and evaluate over-fitting of the calibration models. The prediction performance of the models were evaluated by correlation coefficients (*R*) of calibration (*R*_*C*_), cross-validation (*R*_*CV*_), prediction (*R*_*P*_), root mean square error of calibration (RMSEC), cross-validation (RMSECV), and prediction (RMSEP)^18^.

### Spatial distribution of leaf pigments

**Figure 6:**
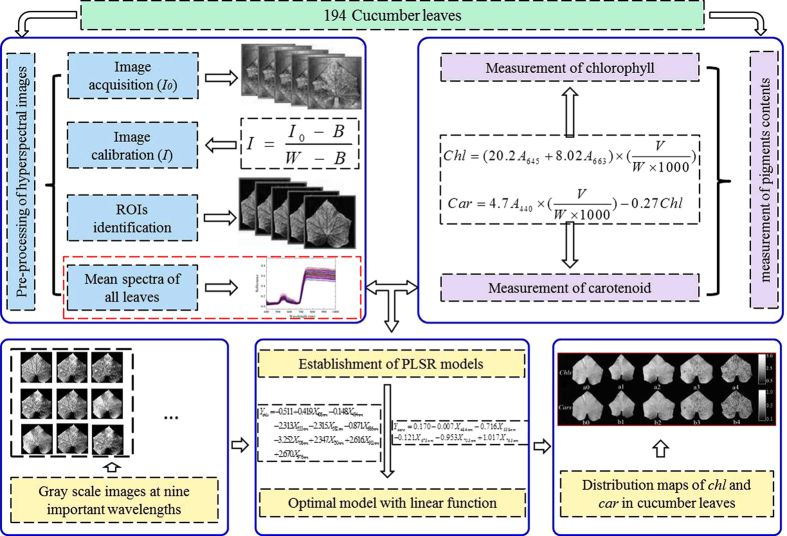
Main steps of determining of *Chl*-HSI and *Car*-HSI values in cucumber leaves infected with ALS by hyperspectral imaging. There were three main steps to map the *Chl*-HSI and *Car*-HSI spatial distribution. (1) After hyperspectral images acquisition and correction, reflectance values were extracted from the regions of interest (ROIs). (2) Then *Chl* and *Car* contents were determined by biochemical analysis (*Chl*-BA, *Car*-BA). Averaged spectra of samples and contents of *Chl*-BA, *Car*-BA were used to select the important wavelengths (IWs) for developing the partial least square regression (PLSR) models. (3) The established PLSR models were used to calculate the *Chl*-HSI and *Car*-HSI values at each pixel on the hyperspectral images. Finally, *Chl*-HSI and *Car*-HSI spatial distribution in cucumber leaves infected with different severities of *Pseudomonas syringae* pv. *lachrymans* infection were displayed with the help of MATLAB software.

Observation of the *Chl* and *Car* content variances in cucumber leaves is helpful to measure the growth conditions of the plants. An optimal calibration model is firstly established with the mean spectra of the samples and their corresponding chemical values^37^. Then *Chl*-HSI and *Car*-HSI contents of each pixel were predicted by inputting the spectrum of each pixel into the optimal regression models. The distribution maps of *Chl*-HSI and *Car*-HSI contents were generated according to the predicted values in the corresponding spatial position. This visualization process was carried out in MATLAB R2009a. Figure. 6 illustrates the main steps of mapping of *Chl*-HSI and *Car*-HSI spatial distribution in cucumber leaves infected with ALS by hyperspectral imaging technique.

## Additional Information

**How to cite this article**: Zhao, Y.-R. *et al*. Hyperspectral Imaging for Determining Pigment Contents in Cucumber Leaves in Response to Angular Leaf Spot Disease. *Sci. Rep.* **6**, 27790; doi: 10.1038/srep27790 (2016).

## Figures and Tables

**Table 1:** Results of analysis by the PLSR models for estimating *Chl*-HSI and *Car*-HSI contents based on the IWs (RC-PLSR) and whole-wavelengths (W-PLSR).

Pigments	Models	N.^a^/LVs^b^	Calibration		Validation		Prediction		Number
			*R*c	RMSEC	*R*cv	RMSECV	*R*p	RMSEP	
*Chl*	W-PLSR^c^	476/7	0.913	0.315	0.894	0.347	0.87	0.255	Model 1
	RC-PLSR^d^	9/5	0.901	0.335	0.889	0.355	0.871	0.25	Model 2
*Car*	W-PLSR	476/2	0.796	0.08	0.787	0.081	0.886	0.049	Model 3
	RC-PLSR	5/1	0.803	0.079	0.793	0.08	0.876	0.05	Model 4

Note: N.^a^: The number of the variables; LVs^b^: Latent variables; W-PLSR^c^: developed PLSR models based on whole wavelengths (476 variables); RC-PLSR^d^: IWs selected by RCs were applied to the established PLSR models (9 and 5 variables).
